# A novel surgical treatment approach for the vertical root fracture of posterior teeth: a case report with 24-month review

**DOI:** 10.1186/s12903-024-04268-9

**Published:** 2024-04-24

**Authors:** Qing Sun, Furong Han, Wei Fan

**Affiliations:** State Key Laboratory of Oral & Maxillofacial Reconstruction and Regeneration, Key Laboratory of Oral Biomedicine Ministry of Education, Hubei Key Laboratory of Stomatology, School & Hospital of Stomatology, Wuhan University, Wuhan, People’s Republic of China

**Keywords:** Vertical root fracture, Resin, iRoot BP plus, Intentional replantation, Posterior teeth

## Abstract

**Background:**

Up to 25% of the tooth extraction after root canal treatment could be attributed to the vertical root fracture (VRF). The treatment choice for teeth with VRF would mostly be the extraction despite some repairing methods were also reported. The repairing treatment result of VRF would mostly depend on the fixation strength and the bioactivity of the repairing materials, especially for the posterior teeth with high masticating stresses. This case report designed a novel surgical treatment approach for the VRF of posterior teeth.

**Methods:**

a maxillary premolar with buccal-palatal complete VRF was treated with a new dual-layered repairing approach using adhesive resin + iRoot BP Plus bioceramic cement to fill the modified fracture line with retention forms through the intentional replantation.

**Results:**

At the 24-month review, the tooth showed desirable periodontal healing and normal function.

**Conclusions:**

This case report indicated that the dual-layered repairing approach might be effective for saving the posterior teeth with VRF. Nevertheless, further clinical trials are needed for its long-term result.

## Background

Vertical root fracture (VRF) is one of the most serious complications for teeth undergone root canal treatment and accounts for about 3.69–25% of the post-treatment tooth extraction [1]. To save the teeth with VRF, tentative efforts have been tried by clinicians to repair the VRF, including using adhesive composite resin, CO_2_ and Nd: YAG laser, or bioceramic materials [2, 3]. Although these reported cases showed promising results within different review time frame, the teeth reported were mostly limited to anterior teeth (incisors and canine) [3, 4].

Adhesive resins were reported to successfully bond the fractures of VRF, but the low bioactivity of resin would be a potential problem for the long-term periodontal healing of teeth with VRF [5]. This problem has been confirmed in the treatment of malformed lingual radicular groove which uses bioceramic cement rather than composite resin to fill the groove as the periodontal pockets along the resin surface could easily recur [5]. On the other hand, calcium silicate-based bioceramic cement, such as MTA, biodentine or iRoot BP plus are desirable for repairing various root canal perforations or resorptions due to its high bioactivity for periodontal tissue attachment and hard tissue regeneration [6–8]. Despite this, bioceramic materials cannot provide strong bonding strength to hold fractures in position [9].

Based on these concerns, a new dual-layered approach using adhesive resin + iRoot BP plus cement through the intentional replantation was designed in this case report as an effective method for the treatment of posterior teeth with VRF.

## Case report

**Fig. 1:**
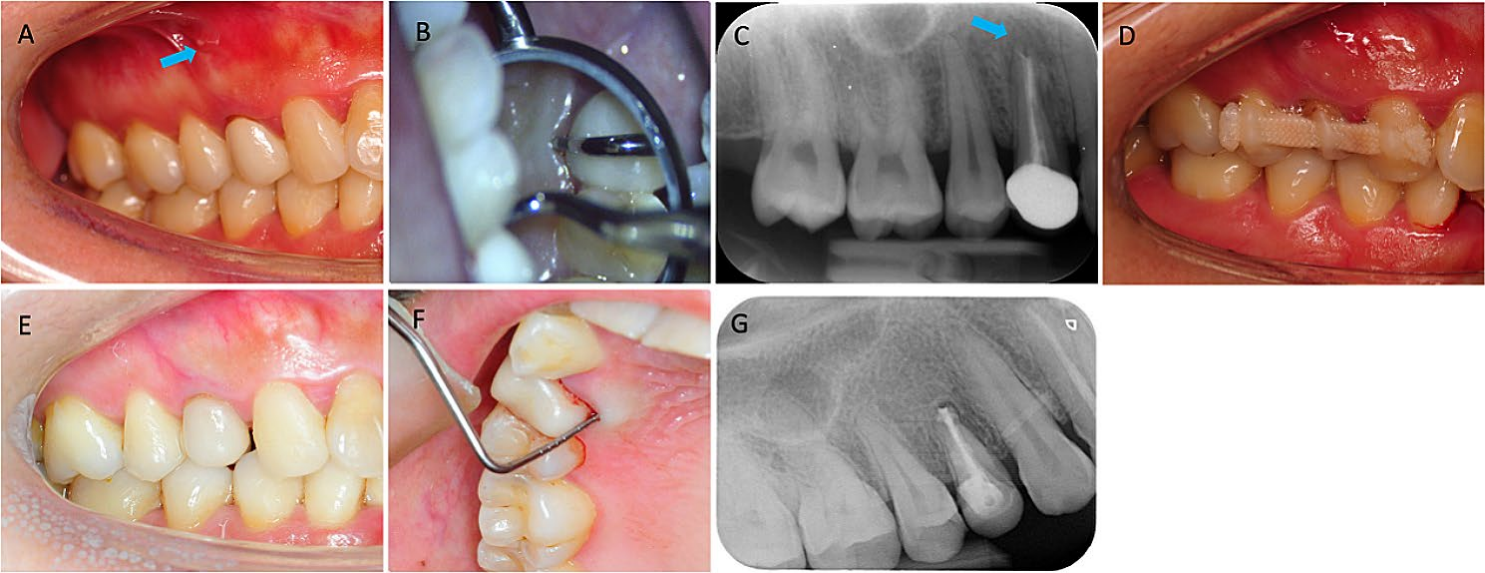
Intra-oral pictures and X-ray images before, after and at 24-month review of surgery. **(A)** Intra-oral picture before surgery showing a sinus track near the apical area of tooth #14(arrow); **(B)** Deep narrow isolated pocket on the palatal side of tooth #14;**(C)** X-ray image before surgery showing a radiolucent area around the apex of tooth #14(arrow); **(D)** Fixation of tooth #14 after the surgery; **(E)** Intra-oral picture at 24-month review showing normal gingiva mucosa;**(F)** Periodontal probing at 24-month review showing about 3 mm probing depth; **(G)** X-ray image at 24-month review showing the healing of periapical defect

**Fig. 2:**
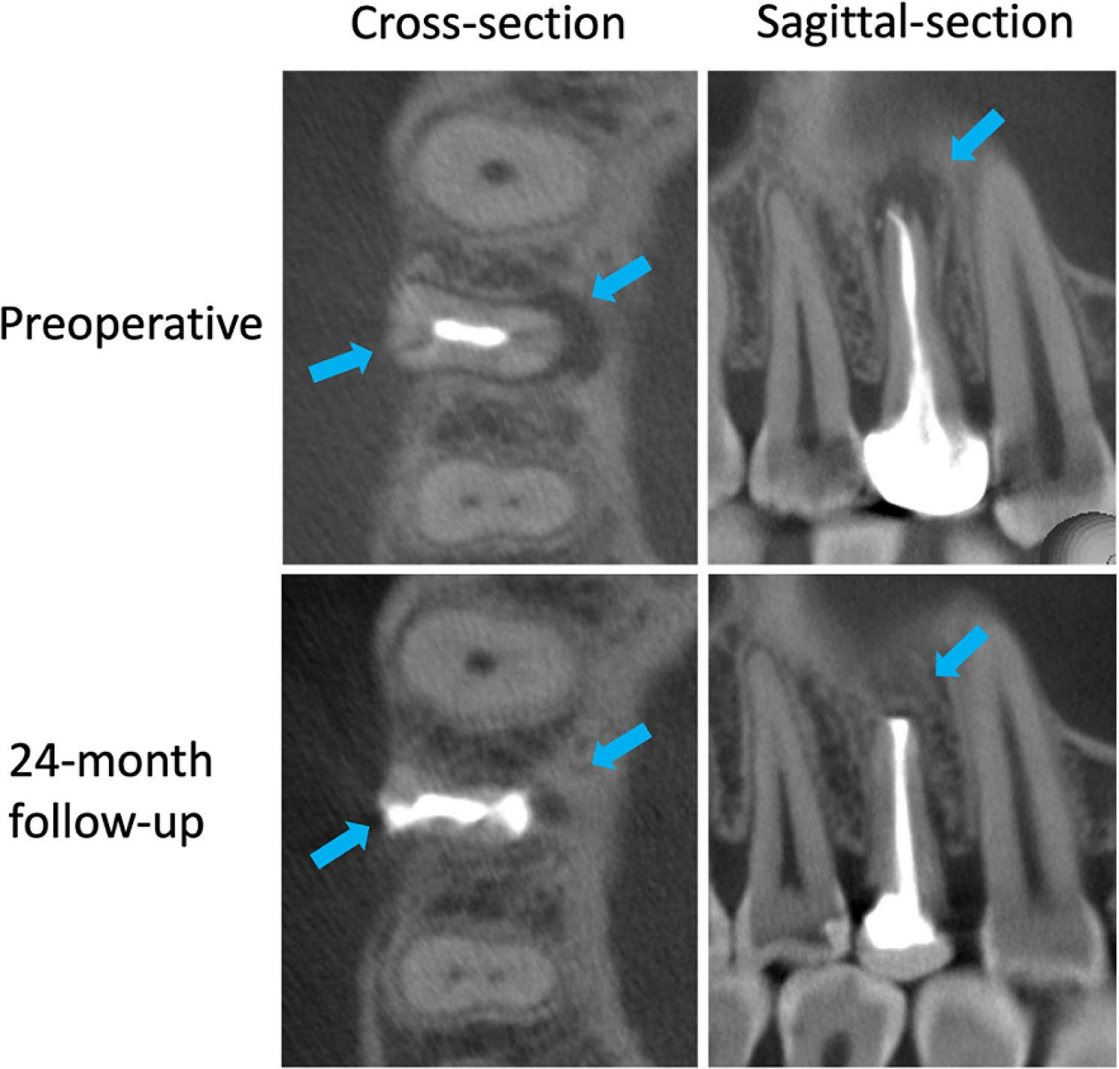
CBCT cross and sagittal images of tooth #14 with VRF before surgery and at 24-month review (arrows showing the root fractures, periodontal defect status or filling materials)

A 34-year-old Chinese female was referred for a gingival pustule of maxillary right posterior teeth. She reported tooth #14 had root canal treatment and a full crown restoration 2 years ago. Intraoral examination revealed a sinus tract on the labial gingival mucosa near the apical area of tooth #14 (Fig. 1A) [10]. The tooth was sensitive to vertical percussion. An 8–10 mm deep narrow isolated pocket was detected on the palatal side of the tooth (Fig. 1B) [10]. Pre-operative radiograph (Fig. 1C) and CBCT images confirmed a large area of bone destruction around the apex and palatal side of the root (Fig. 2) [10]. Besides, vertical buccal-palatal fracture lines on both buccal and palatal sides of the root were identified on CBCT images (Fig. 2) [10]. Based on the examinations, tooth #14 was diagnosed as VRF.

**Fig. 3:**
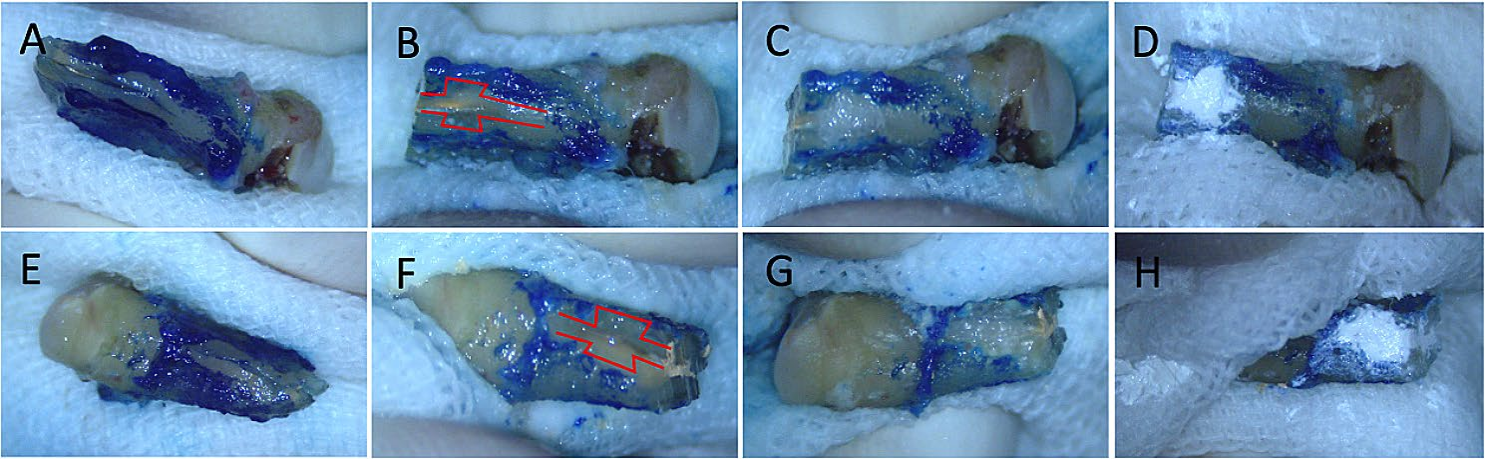
Repairing procedures of vertical root fractures (microscopic view at about ×10 magnification). **(A, E)** Vertical root fractures on buccal and palatal side of tooth #14 (stained with with methylene blue); **(B, F)** Expanding the fracture and preparing the trapezoidal retention forms on both sides of the fracture; **(C, G)** Filling the fracture with adhesive resin on buccal and palatal sides of tooth #14; **(D, H)** Covering the resin surface with iRoot BP Plus on both sides of tooth #14

**Fig. 4:**
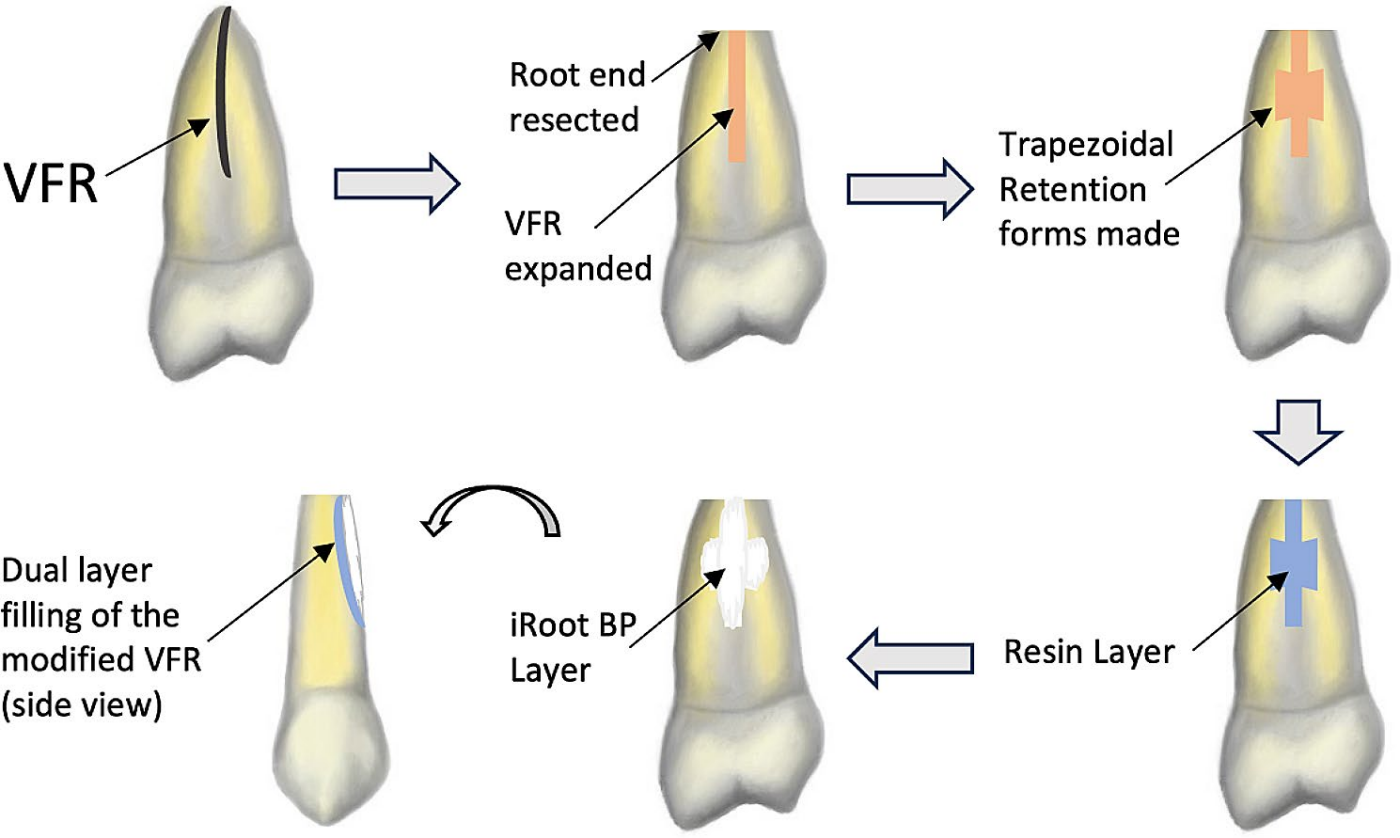
Schematic illustration of surgical procedures

The new surgical procedures for the VRF were shown and illustrated in Figs. 3 and 4. Patient was given local anesthesia with a 2% articaine solution containing 1:1000 epinephrine (Zorcaine, Acteon Pharma, France). The affected tooth was then gently extracted and wrapped in gauze saturated with sterile normal saline (Fig. 3) [10]. The root surface was stained with methylene blue and the fracture was identified under a microscope (Zumax Medical Co. Ltd., Suzhou, China) (Fig. 3A, E) [10]. A high-speed handpiece (about 7000r/s) was used to resect 3 mm of root apex and expand the fractures to a width of about 1 mm and a depth of 1.5–2 mm (Figs. 3 and 4). Two trapezoidal retention forms were prepared on both sides of the fracture line (Fig. 3B, F, with 1 mm width and 1.5–2 mm depth) [10]. Sterile normal saline was used as the coolant during the operation. In the meantime, a 3 mm apical canal cavity was retrograde prepared using an ultrasonic tip. The expanded fractures and the retention forms were filled with self-etching light-curing adhesive resin (Ketac Molar Easymix; 3 M ESPE, St Paul, MN) (Fig. 3C, G) [10]. After that, about 0.5–1 mm thick surface resin was removed. The resin surface together with the apical canal were filled with iRoot BP Plus cement (Innovative Bioceramix Inc., Vancouver, Canada) (Fig. 3D, H) [10]. The repaired teeth were then carefully replanted and elastically fixed with a fiber band (Kuraray, Shanghai, China) for 4 weeks(Fig. 1D) [10]. All these procedures were finished within 15 min.

At the 24-month review, the X-ray radiograph (Fig. 1G) and CBCT revealed a significant periodontal bone regeneration (Fig. 2). The tooth was asymptomatic with normal gingiva mucosa, about 3 mm periodontal probing depth and normal mobility (Fig. 1E, F).

## Discussion

Previous case reports about the treatment of VRF used either the composite or bio-ceramic materials to simply fill the fracture mostly of anterior teeth [3, 4]. In this case report, a novel dual-layered repairing approach using both composite resin and bio-ceramic materials was designed for the VRF of posterior teeth. According to the treatment result of this case report, the dual-layered repairing approach seems effective within the 24-month review period. The following three factors could partially explain the treatment result: (i) Although adhesive resin shows high dentin bonding strength, failures did occur in premolars and molars treated with adhesive resin due to the high masticating stresses [3]. To avoid this, small trapezoidal retention forms were added to both sides of fracture to enhance the fracture fixation. (ii) Although tooth replantation has been used in clinic for decades and shows a high success rate [11, 12], the periodontal healing ability is still the determining factor for the treatment result of tooth replanation. Resin alone cannot heal the periodontal defects due to its limited bioactivity for the regeneration of periodontal tissues [5]. The iRoot BP Plus cement has ideal bioactivity and can induce the regeneration of periodontal hard tissues [5]. In this report, to achieve better healing of periodontal defects, the iRoot BP Plus cement layer was added on the resin surface. (iii) The operation time was controlled within 15 min and the root was kept moist during the surgery procedures [5]. Periodontal ligament (PDL) is the most important issue for the periodontal healing. To minimize damages to the PDL, the extraoral operation time should be kept as short as possible. Extraoral time more than 30 min would increase the chance of post-operative surrogate root resorption. In this report, at the 24-month review, the periodontal probing depth returned to the 3 mm normal depth, indicating the healing of PDL tissues.

## Conclusions

Based on this case report, the dual-layered approach could be an effective method to preserve the posterior teeth with VRF. Nevertheless, further clinical trials are needed for its long-term result.

## Data Availability

No datasets were generated or analysed during the current study.
